# Supplementary material for: The causal role of circulating inflammatory markers in osteoporosis: a bidirectional Mendelian randomized study
Source: Front Immunol. 2024 Jul 18;15:1412298. doi: 10.3389/fimmu.2024.1412298 (PMC11291241; doi:10.3389/fimmu.2024.1412298)
Supplement: Supplementary file 1 [file DataSheet_1.zip › supplementary material/CIM-OSTEOPOROSIS /GCST90274784.csv_forest.pdf]

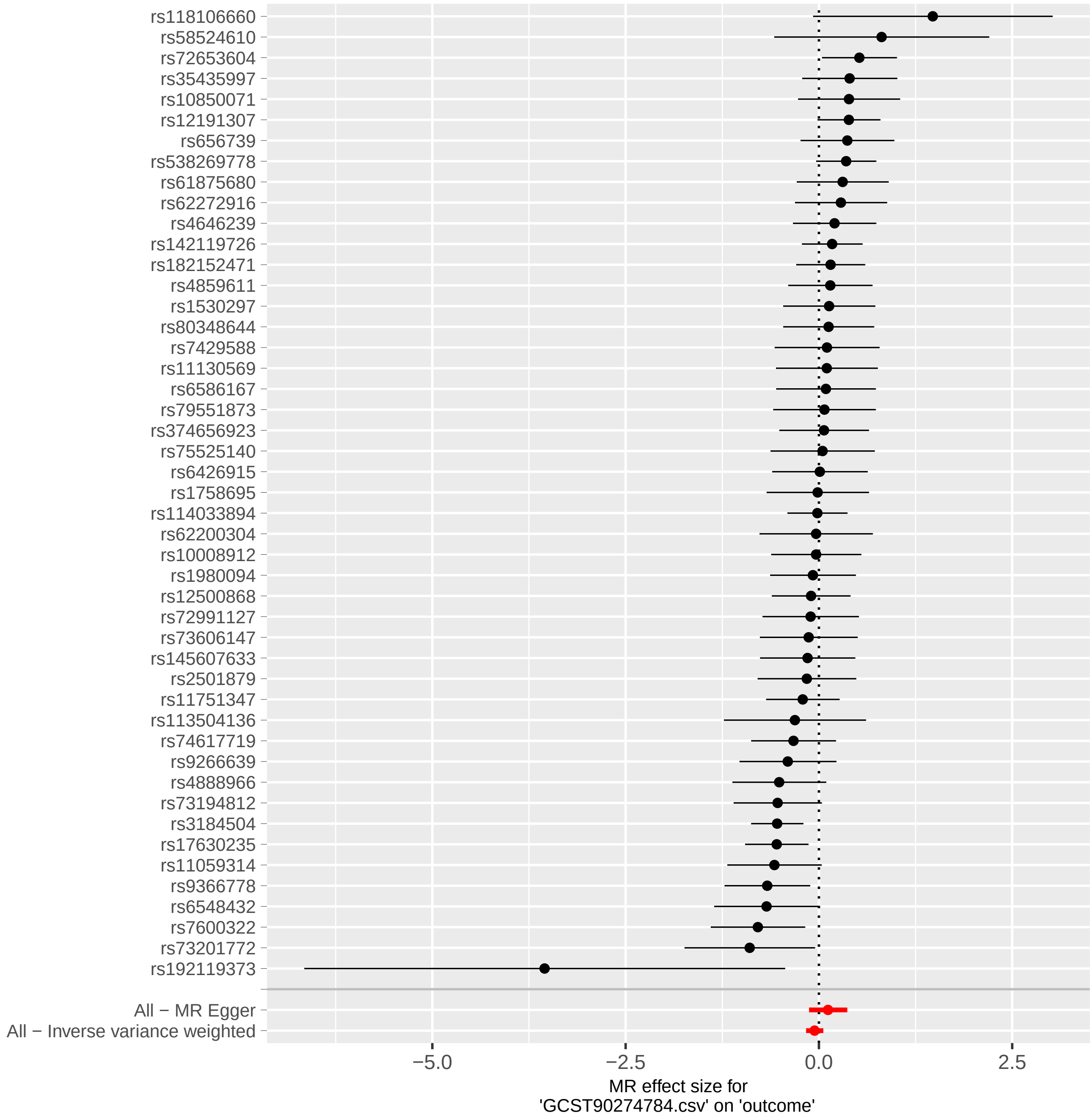
rs118106660
rs58524610
rs72653604
rs35435997
rs10850071
rs12191307
rs656739
rs538269778
rs61875680
rs62272916
rs4646239
rs142119726
rs182152471
rs4859611
rs1530297
rs80348644
rs7429588
rs11130569
rs6586167
rs79551873
rs374656923
rs75525140
rs6426915
rs1758695
rs114033894
rs62200304
rs10008912
rs1980094
rs12500868
rs72991127
rs73606147
rs145607633
rs2501879
rs11751347
rs113504136
rs74617719
rs9266639
rs4888966
rs73194812
rs3184504
rs17630235
rs11059314
rs9366778
rs6548432
rs7600322
rs73201772
rs192119373
All − MR Egger
All − Inverse variance weighted
−5.0
−2.5
0.0
2.5
MR effect size for
'GCST90274784.csv' on 'outcome'
